# Checkpoint Inhibitor-Induced Colitis: An Update

**DOI:** 10.3390/biomedicines11051496

**Published:** 2023-05-22

**Authors:** Giuseppe Losurdo, Daniele Angelillo, Nicolas Favia, Maria Chiara Sergi, Alfredo Di Leo, Giacomo Triggiano, Marco Tucci

**Affiliations:** 1 Section of Gastroenterology, Department of Precision and Regenerative Medicine and Ionian Area, University of Bari “Aldo Moro”, 70124 Bari, Italy; 2 Medical Oncology Unit, Azienda Ospedaliero Universitaria Policlinico di Bari, 70124 Bari, Italy; 3 Department of Interdisciplinary Medicine, University of Bari “Aldo Moro”, 70124 Bari, Italy

**Keywords:** immunotherapy, cancer, immune checkpoint inhibitors, colitis, diarrhea

## Abstract

Immunotherapy with immune checkpoint inhibitors (ICIs) nowadays has indications for several solid tumors. The current targets for ICIs are CTLA-4, PD-1, and PD-L1 receptors. Despite the clinical advantages derived from ICIs, a variety of side effects are linked to overstimulation of the immune system. Among these, ICI-related colitis is one of the most common, with a disabling impact on the patient. Diarrhea, abdominal pain, abdominal distension, cramping, and hematochezia are the most common ICI enterocolitis presenting symptoms. The most frequently used grading system for assessment of the severity of ICI enterocolitis is called the Common Terminology Criteria for Adverse Events (CTCAE) grading. With regard to the histological picture, there is no specific feature; however, microscopic damage can be classified into five types: (1) acute active colitis, (2) chronic active colitis, (3) microscopic colitis-like, (4) graft-versus-host disease-like, and (5) other types. Supportive therapy (oral hydration, a bland diet without lactose or caffeine, and anti-diarrheal agents) is indicated in mild colitis. Symptomatic treatment alone or with loperamide, a low-fiber diet, and spasmolytics are recommended for low-grade diarrhea. In more severe cases, corticosteroid treatment is mandatory. In refractory cases, off-label use of biological therapies (infliximab or vedolizumab) was proposed.

## 1. Introduction

### 1.1. General Principles of Immunotherapy and Immune Checkpoint Inhibitors (ICIs)

Nowadays, due to several immunological findings in the field of oncology, immunotherapy (IT) has made a change to cancer treatment. Notwithstanding the fact that chemotherapy and radiotherapy still remain the first therapeutic options in some malignancies, results of immunotherapy in melanoma, renal cell carcinoma, lung cancer, colorectal cancer, gastric cancer, and others have definitely revolutionized and expanded their therapeutic landscape [1]. The T cell activation mechanism, apart from the binding of the major histocompatibility complex (MHC) by dendritic cells (DCs) and T cell receptors (TCR), requires the co-stimulation or co-inhibition signaling determined by immune checkpoints. Thus, ICIs seem to be a promising strategy in terms of therapeutic response. The current targets for ICIs are CTLA-4 and PD-1 and PD-L1 receptors [2].

The anti-CTLA-4 monoclonal antibody (mAb) ipilimumab was the first ICI approved by the US Food and Drug Administration (FDA) in 2011 for the treatment of advanced melanoma. CTLA-4 is a membrane receptor protein expressed by cytotoxic T lymphocytes that, when bound to proteins presented by DCs such as B7-1 or B7-2, leads to inhibitory signals in lymphocytes. The anti-CTLA-4 mAb, therefore, prevents inhibitory signaling on cytotoxic T-cells and enhances the immune response against cancer cells [3].

Similarly to CTLA-4, PD-1 is an inhibitory membrane receptor protein of cytotoxic T lymphocytes that binds to the PD-L1 and PD-L2 expressed by many cells, such as DCs and cancer cells. Thus, ICIs blocking PD-1 and PD-L1 prevent the binding between these two proteins by enhancing the immune response [4].

In current clinical practice, mAbs anti-PD-1 are pembrolizumab, nivolumab, and cemplimab, while mAbs anti-PD-L1 are atezolizumab, avelumab, and durvalumab.

Interestingly, ICIs can also be combined, as is the case of ipilimumab and nivolumab for the treatment of several tumors such as lung, kidney, and melanoma [5,6,7], as well as tremelimumab and durvalumab, which have recently been approved for the treatment of hepatocellular carcinoma [8]. Considering the indisputable success that ICIs have obtained in some tumors, new emerging targets such as LAG3, TIM3, TIGIT, CD39, B7H3, CD47, CD73, and adenosine A2A receptor are being investigated. Although encouraging results are already available in preclinical studies and clinical trials relating to the aforementioned molecules, there is still no drug that has entered into clinical practice [9].

### 1.2. Immuno-Related Adverse Events (irAEs) and ICI Colitis

Despite the clinical advantages derived from ICIs, a variety of side effects are linked to overstimulation of the immune system. In this context, the development, severity, and underlying biology of irAEs may differ [10]. Immune-related adverse events (IrAEs) generally arise within weeks to months after treatment initiation (a median onset of approximately 40 days) and may be defined as “acute” if they develop during treatment, “delayed” if they appear following ICI completion, and “chronic” if they persist beyond 12 weeks after discontinuation of therapy [11]. The occurrence of irAEs depends on the agent, dose, tumor type, disease setting, and, obviously, patient characteristics. Indeed pre-existing autoimmune illnesses may be predisposing toward irAEs [12].

Although any organ system can be affected by irAEs, skin toxicities (non-specific maculopapular rashes, psoriasis, and lichenoid reactions) are the most common ones, but rarely affect the treatment schedule. Instead, pneumonitis and myositis can be life-threatening events, like myocarditis, pericarditis, vasculitis, acute coronary syndrome, and conduction disease. On the other hand, less frequent toxicities that usually do not impair treatment continuation include arthritis, polymyalgia, and endocrinopathies, which could lead to persistent sequelae with life-long replacement and/or symptomatic treatments [13].

Herein we reviewed the clinical, endoscopic, and pathogenic features of colitis, which could be one of the leading causes of hospitalization and quality of life deterioration during ICI treatment.

Regarding the pathogenesis of ICI colitis, the systemic mechanism underlying the onset of adverse events is related to CTLA4 expression by regulatory T cells (Treg), and to the binding of CD28 and CD80/86 (on the T cell surface and antigen-presenting cell surface, respectively), an activating signal for T cells. CTLA-4 has a higher affinity for CD80/86 than CD28: when CTLA-4 binds CD80/86, it results in an inhibition of the immune response. In addition, the production of immunosuppressive cytokines such as IL-10 and TGF-b increases the inhibition of the immune system. Instead, PD-1/PD-L1 binding suppresses the production of transcription factors (such as Foxp3), enhancing the immunosuppressive ability of Treg, which maintains immune tolerance and leads to tumor immune escape. ICI (anti-PD-1 and anti-CTLA-4) can therefore reduce Treg activity and increase the expression of cytotoxic T lymphocytes and inflammatory cytokines, triggering an autoimmunity response [2,3].

Studies of translational research showed an abnormal activity of “resident memory CD8+T cells” (TRM CD8+) in mucosal tissues, which express CD69 and CD10 and influence mucosal immunity by recruiting CD8 and CD4 T cells from general circulation. In ICI colitis, TRM CD8+ cells differentiate into cytotoxic T lymphocytes, capable of releasing interferon-gamma (IFNγ) with consequent damage to the intestinal epithelial barrier [14].

Since TMR CD8+ populate the mucous membranes, we can presume why colitis is a common and early adverse event during immunotherapy. Furthermore, the increase in the concentration of these lymphocytes in loco could explain the sudden response to the corticosteroid, which notoriously induces apoptosis of activated T lymphocytes [15]. Additionally, in the context of immune suppression during ICI colitis, an inhibited Treg function and a higher percentage of Th17 cells are described [14,15]. In particular, Th17 cells secrete interleukin (IL)-17, a cytokine increased in colonic biopsies in ICI colitis. Elevated serum levels of interleukin (IL)-17 emerged from a trial evaluating circulating cytokines, chemokines, and growth factors and the outcome of patients with locally advanced melanoma receiving neoadjuvant ipilimumab. These findings highlighted a link between grade 3 diarrhea or colitis and IL-17 levels [16]. Moreover, current evidence suggests an association between colitis and inflammatory macrophages. Macrophages produce Tumor Necrosis Factor-Alpha (TNF-alpha), IL-6, CXCL1, and CXCL9/10 chemokines, thus resulting in the recruitment of additional T cells within the colon and exacerbation of colitis. Furthermore, through chemokine relapse, Th17 cells and macrophages led to neutrophil attraction, increasing inflammation of the gut [16].

Moreover, several clinical trials have investigated potential risk factors such as age, ethnicity, body mass index, genetics, lack of vitamin D supplementation, and gut microbiota composition [10]. The latter plays a primary role in the onset of colitis. In inflammatory bowel disease, the gut microbiome is rich in pro-inflammatory bacteria, such as Proteobacteria, while there is a reduction in *Faecalibacterium prausnitzii*, which has anti-inflammatory properties. The latter is capable of seizing regulatory T cells (T reg) in the intestine. Ipilimumab inhibits regulatory T-cells (Tregs) as an effect of the high expression of CTLA4, and in parallel, increases the activity of effector T cells resulting in tumor response as well as colitis [17]. Furthermore, a prospective study on colitis in patients treated with ipilimumab for melanoma discovered that patients resistant to ICI-induced colitis had higher concentrations of Bacteroidetes bacteria, underlining the function of microbial factors [18]. Research efforts will be directed towards microbiome modulation to avoid irAEs.

## 2. Epidemiology

Bloody diarrhea, mucorrhea, vomiting, fever, fatigue, or weight loss are the hallmarks of colitis: it is estimated that 54% of patients treated with anti-CTLA4 mAbs and the combination of anti-CTLA4 and anti-PD-1 experienced diarrhea, more frequently than anti-PD-1 monotherapy patients (with an incidence of 19%) [19]. As reported by the ESMO Clinical Practice Guideline for diagnosis, treatment, and follow-up of immunotherapy toxicities [20], colitis occurs in 10% of patients who received anti-CTLA4 and in 15% of those receiving a combination,

Indeed, ICIs can cause either mild, moderate, or severe forms of colitis [21]. Among them all, CTLA4-inhibitors and, in particular, ipilimumab, are more frequently associated with colitis; this finding could be explained by the fact that the pathway induced by CTLA-4 is dependent on antigen-presenting cells; therefore, it is upstream compared to the PD1-PD-L1 signal. Therefore, the suppression of Treg induced by ipilimumab is less targeted. Nielsen et al. [22] reported 11.2–4.9% incidence of mild and severe colitis, respectively, using ipilimumab, vs. 1.2–0.2% incidence using PD-1 inhibitors and 0.3–0.004% incidence using PD-L1 inhibitors. Among PD-1 inhibitors and PD-L1 inhibitors, the incidence of mild colitis is more frequent using PD-1 than PD-L1 (1.2% vs. 0.3%, respectively); for severe colitis, there is no significant difference between PD-1 and PD-L1 inhibitors [22]. When PD-1 inhibitors and CTLA4 inhibitors are used in combination for renal cell carcinoma, colorectal cancer, melanoma, small cell lung cancer (SCLC), and hepatocellular carcinoma, the incidence of colitis is higher for a 3 mg/kg dosage of ipilimumab and a 1 mg/kg dosage of nivolumab than a 1 mg/kg dosage of ipilimumab and a 3 mg/kg dosage of nivolumab [23,24]. Microscopic colitis can occur during ICI therapy; intestinal perforation and death are both reported while using ICIs following ipilimumab treatment [25]. Specific risk factors for the development of ICI enterocolitis are still not well established. Further studies are needed to clarify the role of genetic factors, host microbiome, and a history of autoimmune disease prior to their onset [26]. For instance, it has been described, in a group of patients with pre-existing autoimmune diseases and, in particular psoriatic arthritis and rheumatoid arthritis, at an incidence of 38%, with 16% of colitis being classified as grade 3 [27]. In another group of 56 patients with concomitant autoimmune disorders (extremely heterogeneous, with rheumatologic, IBD, and neurologic involvement), there was an onset of colitis in 38% of patients, and 10% had a grade 3 reaction [27].

The risk increases in patients with a previous diagnosis of inflammatory bowel disease (IBD); for instance, the case of a patient with ulcerative colitis was described, who developed severe fulminant colitis after starting atezolizumab treatment for small cell lung cancer [28]. Furthermore, the prevalence of IBD exacerbation following ICI was 36.8% amongst 19 patients with IBD. Patients with exacerbations had more gastrointestinal-related hospitalizations [29]. It has also been established that chronic proton pump inhibitor use for at least 8 weeks is a risk factor for ICI colitis in patients with renal cell carcinoma [30]. The most dangerous complication consists of intestinal perforation (in approximately 1% of patients with colitis). Recently, the association of PD-1/PD-L1 mAbs and chemotherapy provoked an increase in early onset diarrhea (within 1–2 weeks of commencement of therapy) as well as the combination of ICIs with antiangiogenic agents targeting the vascular endothelial growth factor (VEGF) [13].

## 3. Clinical Presentation, Diagnosis, Endoscopic and Histopathological Findings

Diarrhea, abdominal pain, abdominal distension, cramping, and hematochezia are the most common ICI enterocolitis presenting symptoms, which can be accompanied by symptoms of upper gastrointestinal inflammation such as dyspepsia, regurgitation, or heartburn. The most frequently used grading system for assessing the severity of ICI enterocolitis is called the Common Terminology Criteria for Adverse Events (CTCAE) grading, version 5 [31], which classifies the intensity of symptoms in five stages, from 1 (mild) to 5 (death) [Table 1]. Grade 1, 2, and 3 of diarrhea differs from the increase in stool over baseline, i.e., less than four, between four and six, and over seven stools/day, respectively. Grade 4 is characterized by life-threatening consequences (intestinal ischemia, necrosis, bleeding, toxic megacolon, perforation, and systemic shock), so in these cases, urgent intervention is indicated.

**Figure 1:**
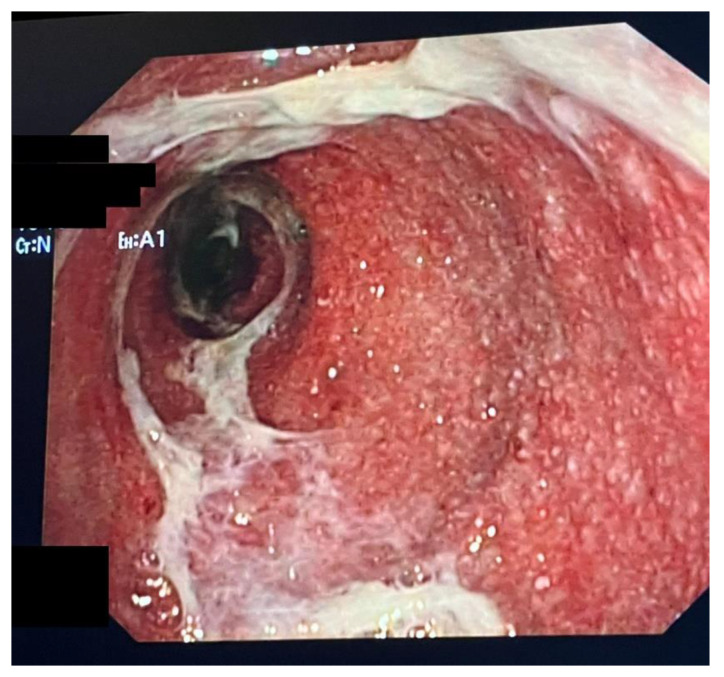
Endoscopic picture of a female patient with melanoma under pembrolizumab therapy who experienced bloody diarrhea. The sigmoid colon appears to be diffusely erythematous, spontaneously bleeding, with small erosions, and covered by a thick layer of mucus. The lumen looks tubular with loss of haustra.

ICI enterocolitis must be suspected when rapid clinical change occurs after a recent administration of these drugs. This type of clinical presentation is more similar to a colonic infection than to inflammatory bowel disease (IBD), so the first step is to carry out laboratory blood tests (complete blood count, metabolic panel, erythrocyte sedimentation rate, C-reactive protein, viral DNA-PCR, tissue transglutaminase IgA, and total IgA) and stool tests (*Clostridioides difficile*, stool cultures, ova and parasites, fecal elastase, and lactoferrin or calprotectin). However, the specificity of these tests is low. Early endoscopy can improve the outcomes of enterocolitis in ICI-treated patients [32]. Even if there are no specific endoscopic patterns of ICI enterocolitis-associated inflammation, endoscopy with biopsy is the gold standard for the diagnosis, and it should be performed before the start of treatment [33,34,35]. Endoscopic findings may vary from normal-appearing mucosa to frank ulcerations, which are the most important factor in predicting how ICI enterocolitis will respond to treatment. In terms of extension, pancolitis is the most common endoscopic pattern [36], but right-sided colitis, isolated gastritis, gastroenteritis, and enteritis are also mentioned. For this reason, a pancolonoscopy is more indicated than a flexible sigmoidoscopy; an example of colitis secondary to ICI is displayed in Figure 1. Esophagogastroduodenoscopy can be considered in some patients. The importance of biopsy, associated with the clinical history of the patient, in particular the recent exposure to ICI, is pivotal to achieving the correct diagnosis, since IC-colitis may frequently mimic IBD, in particular ulcerative colitis.

Diagnostic imaging techniques such as computed tomography (CT) or magnetic resonance imaging (MRI) have a limited role in diagnosing ICI enterocolitis; they are typically helpful only for complicated cases of ICI enterocolitis that could have several intraprocedural endoscopic risks.

Histopathologic grading of ICI enterocolitis is not well established, but IBD scoring indexes can be adapted, with some limitations [37,38,39]. Regarding ICI enterocolitis histological patterns, there is no specific feature; however, microscopic damage can be classified into five types: (1) acute active colitis, (2) chronic active colitis, (3) microscopic colitis-like, (4) graft-versus-host disease-like, and (5) other types (i.e., mixed type, ischemic colitis-like, and non-specific inflammatory reactive changes). The histological report of patients with ICI colitis encompasses the description of the severity of structural/architectural change in the glands, chronic inflammatory infiltrate, lamina propria neutrophil infiltrate, epithelial neutrophils, crypt abscesses, crypt destruction, and erosions/ ulcerations. The significance of basal plasmacytosis and lamina propria eosinophils, which are typical hallmarks of IBD diagnosis, is unclear. On the other hand, apoptosis could be considered an important estimation of histological activity, and an international panel of pathologists considered it appropriate; it can be scored by counting the number of apoptotic bodies in 10 consecutive crypts. Additionally, withered crypts with apoptosis and/or necrotic debris were evaluated as an appropriate measure for scoring. Another feature is surface intraepithelial lymphocytosis: the stratification of infiltration as 0 to 4, 5 to 20, and >20 lymphocytes per 100 colonocytes could be appropriate for the histological assessment of colitis [40,41,42]. Acute active colitis has characteristics comparable to acute infective colitis, such as neutrophilic or eosinophilic infiltration, whereas chronic active colitis is reminiscent of inflammatory bowel disease histological patterns such as crypt abscesses and cryptitis with intraepithelial neutrophils [41,43], even if Adler et al. [44] showed an increased apoptosis index in ICI enterocolitis.

## 4. Therapy and Management

### 4.1. First-Line Management

As mentioned above, the US National Cancer Institute’s Common Terminology Criteria for Adverse Events (CTCAE) classifies toxicity on a scale of 1 to 5 in ascending order of severity; therefore, it is a pivotal item for customizing a strategy for the management of side effects. Furthermore, it is required to prevent treatment discontinuation, complications, and in some cases, death (Table 1) [13].

Stool tests can help to rule out enteric infection, and, in this case, symptoms regress typically within 7–10 days with supportive therapy (oral hydration, bland diet without lactose or caffeine, and anti-diarrheal agents). Symptomatic treatment alone with loperamide, a low-fiber diet, and spasmolytics is recommended for mild diarrhea (CTCAE grade 1) because the use of immunosuppressants has not proved effective. Management is directed at continuing ICI in the absence of other irAEs [10].

In case of symptoms of grade ≥ 2, stopping ICI treatment is essential, and oral corticosteroid therapy (1–2 mg/kg) is mandatory. After the failure of this strategy, intravenous methylprednisolone may be considered with an evaluation of the response every 3–5 days [15]. For mild symptoms, ICI therapy may be continued [13,45,46]. If symptoms persist or evolve to grade 2, endoscopic evaluation may be required in order to classify inflammation into mild or moderate/severe [47,48]. According to AGA guidelines, ICI enterocolitis typically responds to high doses of systemic glucocorticoids. The recommended dose is 0.5–2 mg/kg prednisone equivalent daily (either oral or intravenous), with tapering in 4–6 weeks [49].

### 4.2. Refractory Colitis

Second-line treatment is required for non-responder patients, patients who have recurrent symptoms during the steroid taper, or after a complete steroid course. AGA guidelines recommend a second-line treatment in patients who do not respond to a high glucocorticoid dose within 72 h of initiation or without a complete response within a week. The second-line treatment, mainly composed of vedolizumab or infliximab, is derived from IBD treatment; doses and scheduling are adapted from Crohn’s disease and Ulcerative Colitis treatment (infliximab 5 mg/kg IV and vedolizumab 300 mg IV, typically at weeks 0, 2, and 6, with a minority of patients who need longer treatment) [50,51]; however, shorter regimens can be used with a case-by-case approach [22]. A rapid response is generally obtained in less than 7 days, with a clinical remission rate of 87% and 88% for infliximab and vedolizumab, respectively [2]. The decision of choosing between the use of infliximab or vedolizumab is based on risk factors, such as malignancy, comorbidities, risk of infection, expected duration of treatment, and other concurrent immune-related adverse events [49]. Patients who do not respond to the initial choice of biologic therapy should switch treatment class from infliximab to vedolizumab or vice versa [51]. A systematic review compared data from several studies about biologic therapy in ICI enterocolitis, including a total of 613 patients. The results were similar between infliximab and vedolizumab (87% vs. 88%, respectively, without any significant difference) [22]. However, vedolizumab has shown some advantages, such as slightly fewer hospitalizations (16% vs. 28%), shorter hospitalizations (10.5 days vs. 13.5 days), and shorter steroid use (35 vs. 50 days), despite longer time to clinical response (17.5 vs. 13 days) [52]. Since vedolizumab acts as an inhibitor of the alpha4-beta7 integrin molecule, it inhibits the homing of lymphocytes in the gut: this could explain why it is so effective in ICI colitis. Infliximab is preferred in patients with severe mucosal disease or debilitating symptoms. In the event of biologic therapy failure, there is no well-established third-line treatment. Some options are fecal microbiota transplantation [53] or other biologic therapies, such as ustekinumab, which promotes the activity of helper T cells 1 and 17 by blocking the interleukin-12/23 receptors [54,55]. Tofacitinib and abatacept, which can interfere with antitumor responses, are further options. Among novel therapeutic strategies for patients with refractory colitis to biological agents, small molecules that have already been approved for ulcerative colitis are a new frontier. For instance, tofacitinib inhibits the JAK-STAT (signal transducer and activator of transcription) pathway involved in innate and adaptive immunity and, therefore, in cancer immune surveillance [56].

A case report from China described the successful use of fecal microbiota transplantation in a patient at the later stage of the disease course, after systemic corticosteroid therapy and biologic therapy (vedolizumab) failure [57]. As proof of the relationship between gut microbial composition and response to irAE treatments, in mice with colitis related to ipilimumab, *Bifidobacterium* administration resulted in clinical benefit with persistent therapeutic efficacy of ICIs [58]. Based on the findings of Wang et al. about fecal microbiota transplantation in ICI colitis, a clinical trial (NCT04038619) is investigating this procedure in patients with genitourinary cancer [59].

Weber et al. suggest that the use of budesonide formulated to be released specifically in the colon may play a role in the management of patients with microscopic colitis from checkpoint inhibitors [60]. Another manifestation of ICI gastrointestinal toxicity is ICI-related celiac disease [61]; in these patients, a therapeutic approach with a gluten-free diet seems to be appropriate, and possibly immunosuppressive therapy (steroids or biologic drugs) for the most severe cases, in addition to nutrient repletion (iron, vitamins, and minerals). Saha et al. showed how the implementation of IBD dietary recommendations in patients receiving ICI treatment may have significant impacts on reducing the incidence and severity of colitis [48]. However, further studies are needed to consider specific diets when initiating ICI therapy to prevent ICI enterocolitis.

Finally, in severe cases, extracorporeal photopheresis and elective colectomy are the last resort [62].

### 4.3. Resuming ICI Treatment

Once symptomatic remission is achieved, the most challenging decision is whether to resume ICI treatment. Each situation is evaluated on an individual basis with a multidisciplinary team. In grade 4 colitis, clearly, resuming treatment is not recommended [13,15].

In comparison to other immunotherapy-treated patients, preliminary results suggest that gastrointestinal toxicities are related to higher survival and treatment outcomes [63].

Interestingly, most data propose a favorable and durable response to immunotherapy in patients with irAEs, associated with a longer overall survival. An interpretation could be that an immune-related event reflects the activation of the immune system against cancer [23]. In addition, a retrospective single-center study demonstrates no significant improvement in clinical outcomes and survival in patients receiving ICI rechallenge after the onset of irAEs, compared to the ICI therapy interruption group [64].

Hence, the idea of not resuming treatment is accepted, especially in moderate-severe irAEs.

For such reasons, further prospective and randomized studies are required. Many of these are still ongoing to identify the safest and most appropriate therapeutic strategy.

Moreover, the risk of recurrent ICI colitis after the reintroduction of immunotherapy is 34%. This risk is higher with anti-CTLA4 antibodies as a re-treatment and is related to the immunosuppressants received at the first manifestation of irAEs [65]. However, the therapy of recurrent ICI colitis is similar to other episodic forms.

Several studies support the use of a biological agent (such as infliximab) during ICI rechallenge to reduce the incidence of recurrent ICI colitis, but it is still an experimental and off-label approach that has not been endorsed in clinical practice [66].

Being able to predict the risk of irAEs could be critical. Some markers correlated to irAEs are TCR (T-cell receptor) diversity, lymphocyte cytosolic protein 1, and adenosine diphosphate-dependent glucokinase, both involved in T-cell activation [67].

As mentioned above, cytokines (IL-17 and IL-6) also play a role in the development of these adverse events and may become markers of toxicity [16,68]. Still, to date, the predictive optimal biomarkers of immunotherapy toxicity are still to be defined.

**Figure 2:**
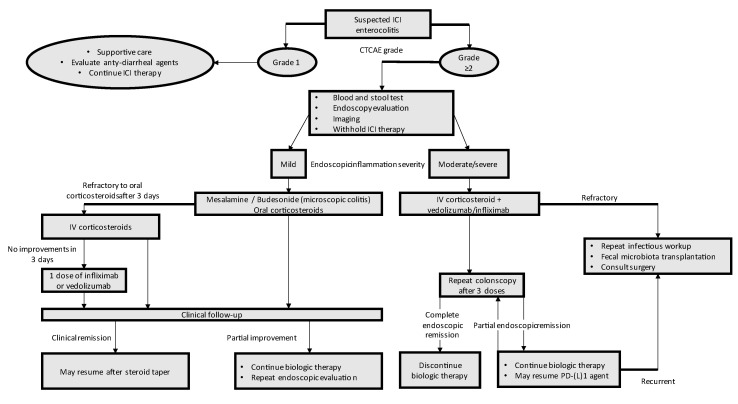
Algorithm for clinical management of ICI colitis, adapted from [45].

An ICI colitis management algorithm is shown in Figure 2 [45].

## 5. Conclusions

The advent of immunotherapy has brought a revolution in the treatment of solid cancers. The discovery that the immunity environment of the cancer can modulate neoplastic cell growth has prompted research to discover molecules (usually mABs) that may block some pathways in the lymphocytes that infiltrate solid tumors [69]. The modulation of the immune system, however, may reflect autoreactive phenomena that engender some side effects [70]. ICI-related colitis is one of these. Most patients may present with self-limiting diarrhea. In other cases, bloody diarrhea with an endoscopic picture of acute colitis mimicking IBD may be observed. Severe cases require hospital admission for proper management, and collaboration between oncologists, gastroenterologists, and endoscopists is crucial. Even the role of the pathologist is relevant, since he/she is often tasked to discriminate between IBD, acute infective colitis, and ICI; stherefore, it is important to provide as much clinical data as possible. The treatment of refractory colitis is a field that still needs clearer indications; biologic therapies derived from IBD, due to clinical and pathogenetic similarities, are still off-label and not standardized. Finally, another issue that requires further investigation is how to resume ICI in order to avoid the recurrence of colitis.

## Figures and Tables

**Table 1:** Common Terminology Criteria for Adverse Events (CTCAE) classification of ICI colitis and enterocolitis.

Variable	Grade 1	Grade 2	Grade 3	Grade 4	Grade 5
Diarrhea	Increase in <4 stools/d over baseline; mild increase in ostomy output compared with baseline.	Increase in 4–6 stools/d over baseline; moderate increase in ostomy output compared with baseline; limiting instrumental ADL.	Increase in ≥7 stools/d over baseline; hospitalization indicated; severe increase in ostomy output compared with baseline; limiting self-care ADL.	Life-threatening consequences; urgent intervention indicated.	Death.
Enterocolitis	Asymptomatic; clinical or diagnostic observation only; intervention not indicated.	Asymptomatic; clinical or diagnostic observation only; intervention not indicated.	Severe or persistent abdominal pain; fever; ileus; peritoneal signs.	Life-threatening consequences; urgent intervention indicated.	Death.

## Data Availability

Not applicable.
